# Irrelevant insights make worldviews ring true

**DOI:** 10.1038/s41598-022-05923-3

**Published:** 2022-02-08

**Authors:** Ruben E Laukkonen, Benjamin T Kaveladze, John Protzko, Jason M Tangen, William von Hippel, Jonathan W Schooler

**Affiliations:** 1 Vrije Universiteit Amsterdam, Amsterdam, The Netherlands; 2 University of California, Irvine, USA; 3 University of California, Santa Barbara, USA; 4 The University of Queensland, St Lucia, Australia

**Keywords:** Psychology, Human behaviour

## Abstract

Our basic beliefs about reality can be impossible to prove and yet we can feel a strong intuitive conviction about them, as exemplified by insights that imbue an idea with immediate certainty. Here we presented participants with worldview beliefs such as “people’s core qualities are fixed” and simultaneously elicited an aha moment. In the first experiment (*N* = 3000, which included a direct replication), participants rated worldview beliefs as truer when they solved anagrams and also experienced aha moments. A second experiment (*N* = 1564) showed that the worldview statement and the aha moment must be perceived simultaneously for this ‘insight misattribution’ effect to occur. These results demonstrate that artificially induced aha moments can make worldview beliefs seem truer, possibly because humans partially rely on feelings of insight to appraise an idea’s veracity. Feelings of insight are therefore not epiphenomenal and should be investigated for their effects on decisions, beliefs, and delusions.

## Introduction

The philosopher Rob Sips^1^ reported how aha moments played an essential role in the manifestation of his psychosis. He described experiencing an “accelerating stream” (p2) of aha moments that revealed the world from so many different perspectives that his worldview simply could not withstand the assault: “This process, in my experiences, was wrecking what I considered to be my “personal worldview”… [the insights] undermined or “derealized” how I looked at things before.” (p3). The idea that aha moments—a sudden feeling of pleasure and certainty that accompanies a new idea—can arise from a change in perspective goes back almost a century^2–4^ and is the basis of how psychologists elicit insight in the laboratory^5^. Like the switching perspectives in the duck-rabbit illusion, aha moments mark a novel discovery in which pre-reflective assumptions change and information is seen in a new light^6^. The experience of Sips^1^ exemplifies the dramatic impact of this process on higher-order worldviews. For Sips^1^, the uncontrolled cascade of aha moments marked the deconstruction of his beliefs, leading to an unstable grasp on reality—like a multidimensional duck-rabbit illusion that will not stop shifting. A recent qualitative study suggests Sips was not alone in this experience^7^.

It is possible that aha moments are simply epiphenomenal, marking but not causing changes in beliefs, much like the steam-whistle of an engine. Under this view, the aha moment is a feeling that correlates with the discovery of a new perspective or solution but has no impact on decision-making or the selection of new ideas^8^. Alternatively, the aha moment may be causally potent^9^. For example, aha moments might provide feedback to the conscious agent about whether an idea is likely to be a good one. Under this view, the aha experience itself partially convinces the agent that the new perspective is true. In other words, the sudden feeling of truth that accompanied the new perspectives Sips was discovering may have exacerbated the destabilizing changes to his beliefs.

Why might Aha! experiences affect belief? Because the processes that precede aha moments can be pre-reflective or implicit^10–18^, aha moments might provide metacognitive information that is not otherwise accessible at a higher-order level. Put differently, the aha moment could plausibly provide information about whether an idea can be trusted in the absence of access to the processes that produced it, much like hunger or fear can signal something important about the state of one’s inner or outer world^19,20^.

There is preliminary evidence that aha experiences can be informative. For instance, aha moments correspond to more accurate solutions to problems^14,21–25^. The correlation between the magnitude of the aha feeling and accuracy has also been assessed in real-time using a measure of grip strength^14^. Results showed that the more tightly participants squeezed the device during the spontaneous aha moment, the more likely it was to be correct. Although these findings do not demonstrate that aha moments are causally agentive, they show that the natural embodiment of the aha correlates with accuracy and may therefore carry valuable information that could be useful for decision-making, similar to the way that hunger carries information about one’s nutritional needs. There is also more direct evidence that aha moments can affect decisions. For example, aha moments that occur when solving anagrams can facilitate false memories, where participants report having seen the word in a list even if they had not^26^. Using a similar paradigm, another study showed that irrelevant aha moments can make mundane facts more believable^9,27^. And finally, ideas accompanied by Aha! experiences are more likely to be remembered^28^, and insights may make it harder to change one’s mind^22^.

In the experiments that follow we test, replicate, and extend the following hypothesis: The seemingly trivial aha moment occasionally elicited by solving anagrams can increase the perceived veracity of important beliefs that serve as the basis of people’s worldviews. We reason that if feelings of insight carry useful information about the quality of the associated ideas, when an aha moment is experienced it might lead to a truer appraisal of the worldview that accompanies it. Metaphorically, the aha moment invokes a "ring of truth", making the temporally coincident but unrelated belief seem valid. In other words, we expect that the participants use their feelings of aha like a heuristic for evaluating the veracity of the associated belief^29^.

## Experiment 1: Demonstration and replication

This experiment was approved by the University of California, Santa Barbara, Human Subjects (ethics) Committee, in accordance with the Declaration of Helsinki.

## Methods

### Design & materials

The experiment had two within subject variables: 2 (Problem: solved or unsolved) × 2 (Aha Experience: present or absent), and one between-subjects factor (Anagrams: present or absent). The dependent measure was truth judgments on a 12-point scale ranging from 1 (definitely false) to 12 (definitely true). We created 15 worldview claims, none of which were objectively demonstrable as true or false. Each claim was constructed such that the last word of it was critical to its meaning (see procedure for an example). The worldview claims were derived conceptually from ‘The Psychology of Worldviews’^30^. We also created 15 anagrams derived from the last word of each claim (see Table S1 in Appendix A for stimuli). Keywords were re-organised into anagrams using a random scramble function, then iteratively pilot tested and adjusted manually until they were neither too difficult nor too easy (approximating 50% solution rates).

### Participants and procedure

**Figure 1:**
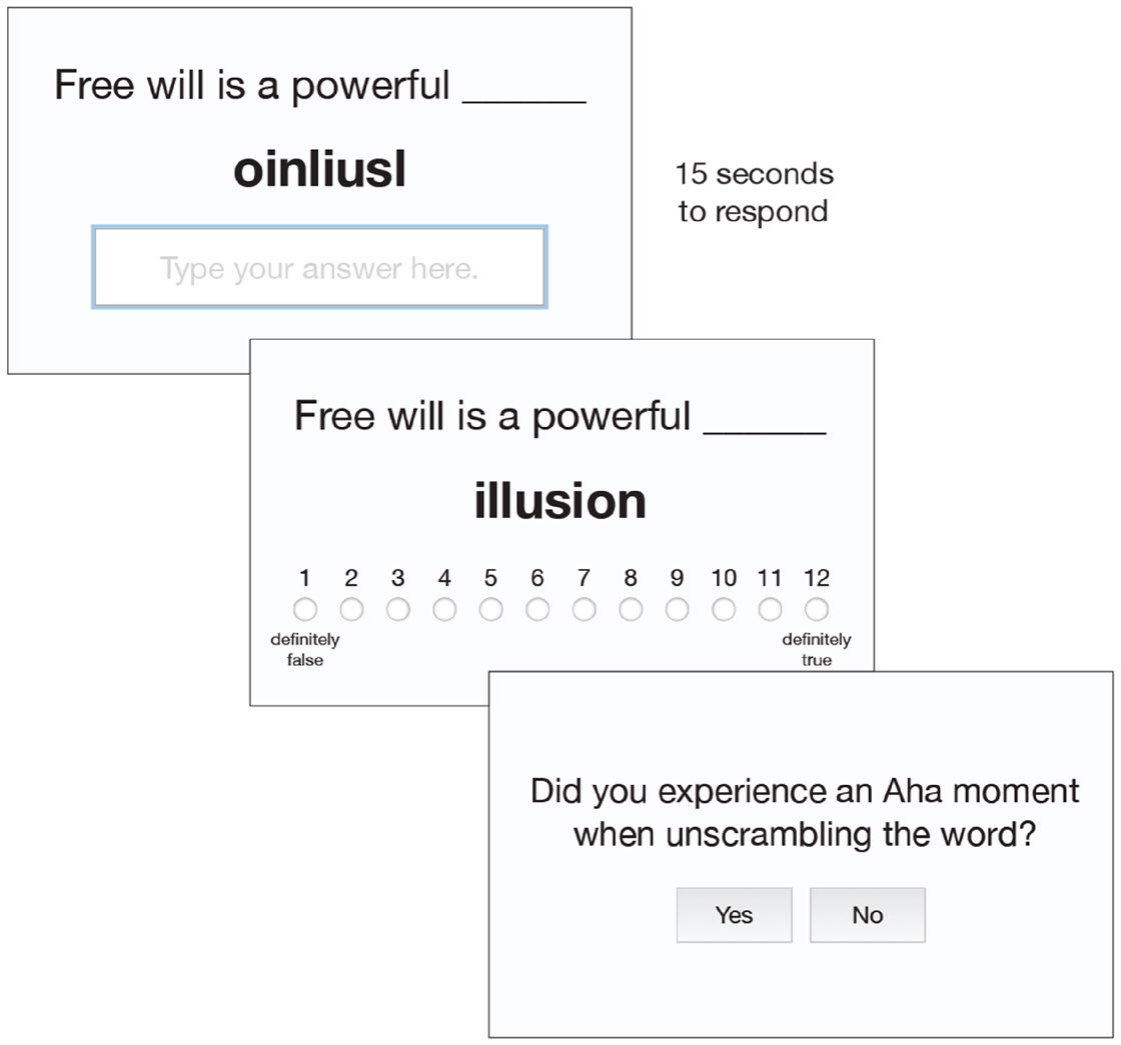
Going from left to right, in each Anagram trial participants were presented with an incomplete claim, for example: “Free will is a powerful ____”. Below it was an anagram that completed the claim (e.g., “oinliusl”). When the answer to the anagram was submitted in the text entry box, or the visible 15-s timer ran out, participants were advanced to the next page. On that page, participants saw the completed claim: “Free will is a powerful illusion” and were asked to make a truth judgment about the claim. Participants then reported whether they experienced an aha moment while solving the anagram. The aha question was presented at the end of each trial so that they would not bias truth judgments, and so that the truth judgments were made as soon as possible following anagram solving while participants were still in the aha state.

This study had two samples of 1500 participants recruited by *Critical Mix* to match the demographics of the U.S (in our samples we had 47% male, 53% female, 0.3% non-binary, with mean age = 51.3, *SD* = 15.7). We used the first sample of 1,500 to test our hypotheses, and then the second 1,500 to assess the replicability of the findings. We conducted a power analysis using the “pwr” package in R (Champely, 2020). Based on the weakest effect from a similar study^9^ (*d* = 0.321) our power analysis showed that we needed 205 participants in each condition to achieve a power of 90%. All participants provided informed consent and were randomly assigned to either the *Anagram* or *No Anagram* (control) condition via written instructions. The procedure for the *Anagram* condition is illustrated in Fig. 1 below. In the *No Anagram* condition, participants were simply presented with the complete propositions (e.g., “It is useless to pursue justice”) and judged how true they were. For a transcript of our description of the Aha! experience, see https://osf.io/7y5af/wiki/home/.

## Results

In our analyses, we rely primarily on two statistical approaches. For between-subjects comparisons, we use Welch’s *t-*tests. For within-subjects analyses, we use multilevel regression models, which account for the hierarchical nature of the data with random intercepts and random slopes for participants and trial numbers^31^. For the multilevel regression models with one binary fixed effect, we present the Cohen’s *d* of the fixed effect^31^. These models assess the influence of the predictors *aha moment* (Y/N) and *correct anagram solution* (Y/N) with truth scores as the DV, as well as the predictor *aha moment* with correct solutions as the DV. We report statistics in accordance with similar previous work^32^. To ensure that our model choices were the best fit for our data, we compared model fit across alternative models using likelihood ratios. One alternate model removed the random slope and random intercept for trial number, and the other alternate model removed the random slope and random intercept for both trial number and participant. We found that for all analyses, our original multi-level models provided significantly better fit (X2s > 154.32, Ps < 0.0001) than models with one or no random effects. Between-subjects analyses were conducted using the *t*-test function within the stats package in R. All within-subjects analyses were conducted using the lmer function within the lme4^33^ package in R, and effect sizes were calculated using the r.squaredGLMM function within the MuMin package. Our data and analysis scripts are available on the OSF (demonstration data: https://osf.io/vqfbu/, replication data: https://osf.io/kf8jx/, complete analyses: https://osf.io/wycmg). For hypotheses and preregistered analysis plans, see the wiki tab on https://osf.io/7y5af for the demonstration and https://osf.io/kdx3u for the replication.

### Demonstration: first 1500 participants

After excluding 247 participants from analysis for failing to solve any anagrams (*N* = 82), solving all anagrams (*N* = 4), experiencing no aha moments (*N* = 178), or experiencing aha moments on all trials (*N* = 23), 1250 participants were included in the analyses. 443 participants were in the *Anagram* condition and 807 were in the *No Anagram* condition (note: we address the differential dropout rate in Experiment 2). On average, participants correctly solved the anagrams 37% of the time (*SD* = 21%), and the anagrams elicited aha moments 36% of the time (*SD* = 22%). Using a multilevel regression model with aha moments as a fixed effect and participants as a random effect, we found that anagrams that elicited aha moments were more likely to be solved correctly (23% solved, *SD* = 18%) than anagrams that did not elicit aha moments (14% solved, SD = 18%), *b* = 0.41, *t* = 16.10, *p* < 0.001, *d* = 0.72.

### Truth judgments: anagrams versus no anagrams (between)

As predicted, a between-subjects Welch’s *t*-test revealed that participants’ average truth scores in the *Anagram* condition were higher (*M* = 6.62, *SD* = 2.09) than participants’ average truth scores in the *No-Anagram* condition (*M* = 5.75, *SD* = 2.05), *t*(781) = 7.54, *p* < 0.001, *d* = 0.46. Overall, the presence of the anagram—including both solved and unsolved anagrams—increased truth judgments regarding the worldview claims.

### Truth judgments: solutions and aha moments (within)

We used a multilevel regression model to test our prediction that claims associated with solved anagrams would be rated as more likely to be true than claims associated with unsolved anagrams, including solving as a fixed effect and participants as a random effect. As predicted, solved anagrams resulted in higher truth ratings (*M* = 7.06, *SD* = 2.70) than unsolved anagrams (*M* = 6.45, *SD* = 2.15), *b* = 0.60, *t* = 4.97, *p* < 0.001, *d* = 0.16. We also used a multilevel regression model to test our prediction that claims would be rated as more likely to be true if they were accompanied by aha moments while solving the anagram, including aha moments as a fixed effect and participants as a random effect. As predicted, participants provided higher truth ratings on trials where they reported experiencing an aha moment (*M* = 7.21, *SD* = 2.73) than on trials where they did not experience an aha moment (*M* = 6.35, *SD* = 2.26), *b* = 0.89, *t* = 6.85, *p* < 0.001, *d* = 0.23. Finally, we examined aha moments among the subset of correctly solved anagrams. Correctly solved anagrams accompanied by aha moments had higher truth ratings (*M* = 7.28, *SD* = 2.92) than correctly solved anagrams without aha moments (*M* = 6.29, *SD* = 2.90), *b* = 0.89, *t* = 5.13, *p* < 0.001, *d* = 0.23.

### Replication: second 1500 participants

After excluding 261 participants from analysis for failing to solve any anagrams (*N* = 123), solving all anagrams (*N* = 1), experiencing no aha moments (*N* = 155), or experiencing aha moments on all trials (*N* = 47), 1239 participants were included in analyses. 434 participants were in the *Anagram* condition and 805 were in the *No Anagram* condition. On average, participants correctly solved the anagrams 36% of the time (*SD* = 21%), and the anagrams elicited aha moments 37% of the time (*SD* = 23%). Using a multilevel regression model with aha moments as a fixed effect and participants as a random effect, we found that anagrams that elicited aha moments were more likely to be correctly solved (*M* = 21%, *SD* = 17%) than anagrams that did not elicit aha moments (*M* = 15%, *SD* = 17%), *b* = 0.35, *t* = 15.25, *p* < 0.001, *d* = 0.62.

### Truth judgments: anagrams versus no anagrams (between)

As predicted and consistent with the first sample, participants’ average truth scores in the *Anagram* condition were higher (*M* = 6.81; *SD* = 2.09) than participants’ average truth scores in the *No Anagram* condition (*M* = 6.22; *SD* = 1.92), *t*(822) = 4.84, *p* < 0.001, *d* = 0.29. As in the first sample, the presence of the anagram—including both solved and unsolved trials—increased truth judgments.

### Truth judgments: solving and aha moments (within)

We used a multilevel regression model to test our prediction that claims associated with solved anagrams would be rated as more likely to be true than claims associated with unsolved anagrams, including solving as a fixed effect and participants as a random effect. As predicted, solved anagrams resulted in higher truth ratings (*M* = 7.09, *SD* = 2.70) than unsolved anagrams (*M* = 6.69, *SD* = 2.24), *b* = 0.49, *t* = 4.51, *p* < 0.001, *d* = 0.13. We also used a multilevel regression model to test our prediction that claims would be rated as more likely to be true if they were accompanied by aha moments while solving the anagram, including aha moments as a fixed effect and participants as a random effect. As predicted, participants provided higher truth ratings on trials where they reported experiencing an aha moment (*M* = 7.34, *SD* = 2.71) than on trials where they did not experience an aha moment (*M* = 6.52, *SD* = 2.23), *b* = 0.91, *t* = 7.58, *p* < 0.001, *d* = 0.25. Finally, we conducted the aha moment analysis among the subset of correctly solved anagrams. Correctly solved anagrams accompanied by aha moments had higher truth ratings (*M* = 7.29, *SD* = 2.96) than correctly solved anagrams without aha moments (*M* = 6.45, SD = 2.74), *b* = 0.82, *t* = 4.53, *p* < 0.001, *d* = 0.23.

### Confirmation + replication

**Figure 2:**
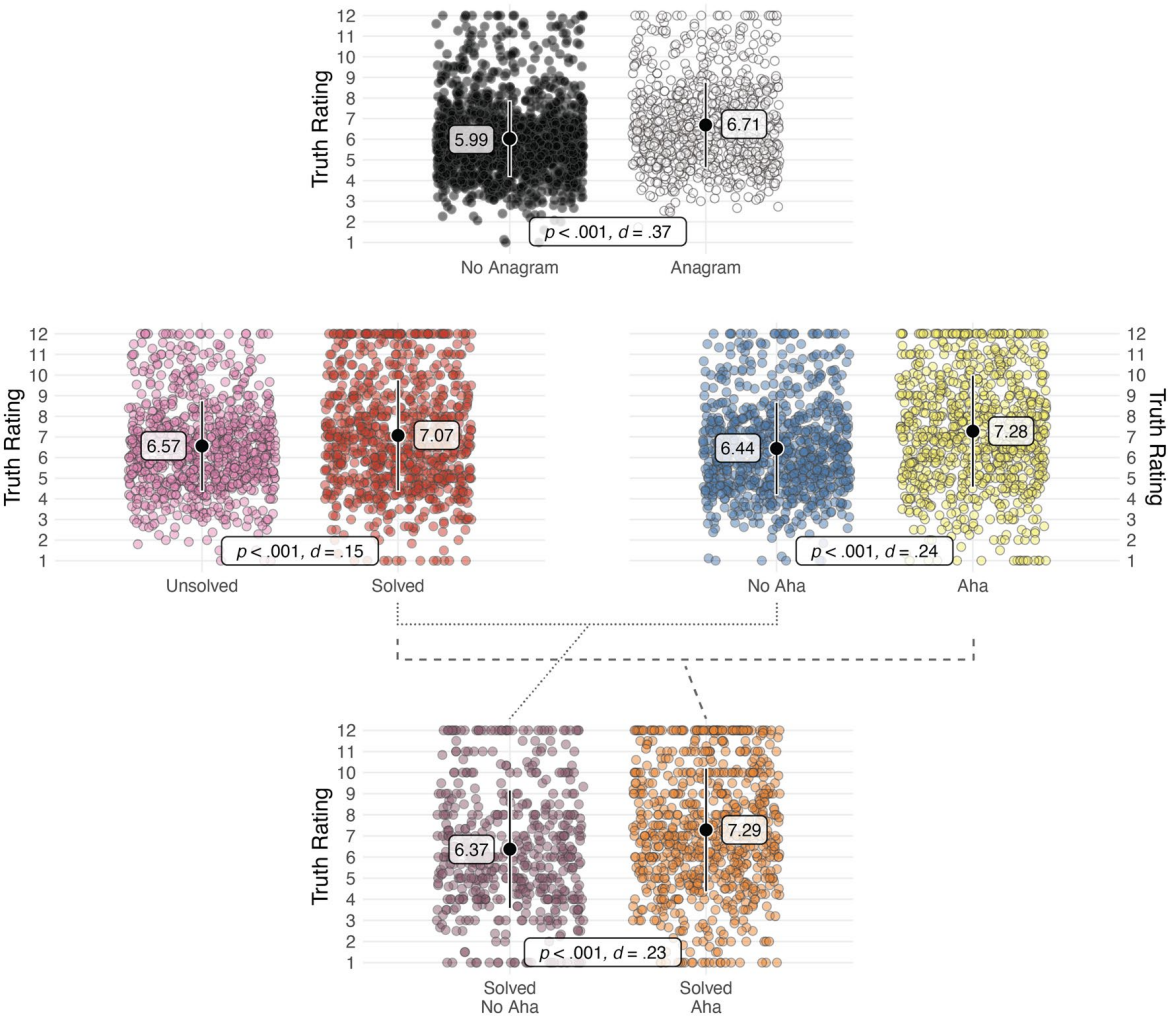
Combined data of the confirmation and the replication, *N* = 2489. The plots (including means and standard deviations) illustrate truth ratings for the different conditions and key comparisons, with *p*-values and effect sizes. Each large black dot represents the mean and black lines represent + /− 1 standard deviation. Circles represent individual participants with a random horizontal jitter to aid visualization.

In Fig. 2, we illustrate the combined results of the confirmation (*N* = 1250) and the replication (*N* = 1239). For each of the illustrated comparisons: *Anagram* (present vs. absent), *Solving* (yes vs. no), and *Aha* (present vs. absent), and aha for solved anagrams (present vs. absent).

### Experiment 2: aha misattribution with delay

This experiment was approved by the University of California, Santa Barbara, Human Subjects (ethics) Committee, in accordance with the Declaration of Helsinki.

## Methods

### Design & materials

In this study we test the assumption that the aha experience needs to be temporally coincident with the claim, by introducing a “delay condition” with a 10-s interval between solving anagrams and being shown claims. Additionally, we address two limitations of the first experiment. First, to reduce differential dropout across conditions, we standardize the difficulty and completion time of each condition. Second, we introduce two new conditions that shift the order of trial components to rule out potential order effects with regard to anagram solving.

The dependent measure was again truth judgments on a 12-point scale ranging from 1 (definitely false) to 12 (definitely true) regarding the 15 claims from Experiment 1 (see Appendix A). To standardize the difficulty, we provided hints when participants attempted to solve the anagrams. Stimuli can be found at https://osf.io/hm6fe/, raw data at https://osf.io/j3m9a/, and analysis code at https://osf.io/bfdgn/.

### Participants and procedure

For this study we had a sample of 1,564 participants recruited by *Critical Mix* to match the demographics of the U.S (our sample was 42.3% male, 57.6% female, 0.1% non-binary, with mean age = 46.8, *SD* = 16.2). Using the pwr function in R, we determined that 99 participants in each of the two key groups of interest would provide sufficient power (0.8) to detect a Cohen’s *d* effect size of 0.4 for the main analysis^34^. Thus, factoring potential dropouts, 1,564 participants randomly assigned to one of four conditions (illustrated in Table 1) should provide more than sufficient power. Participants provided written consent and then were provided written instructions. In this experiment, we included hints alongside anagrams to achieve comparable solving rates for all the conditions. A ten-second delay was provided at the end of each trial.

**Table 1:** Four independent conditions in Experiment 2.

Anagram Normal *N* = 149	Anagram After delay *N* = 157	Anagram After truth *N* = 132	Anagrams After everything *N* = 121
(1) Presentation of an incomplete proposition and concurrently resolving an anagram that completes the proposition (with the help of a hint) within 20 s or presentation of the solution after 20 s if no response is submitted (2) A truth judgment about the completed proposition (3) Reporting whether an aha moment occurred Aim: This is the experimental condition from Experiment 1	(1) Resolving an anagram with the help of a hint within 20 s or presentation of the solution after 20 s if no response is submitted (2) A 10-s "delay" period (3) A truth judgment about the completed proposition (4) Reporting whether an aha moment occurred Aim: This condition tests whether the aha moment needs to be temporally coincident with the claim	(1) Making a truth judgment about a completed claim (2) Unscrambling the keyword from a *different* claim with a hint (3) Reporting whether an aha moment occurred Aim: This condition establishes baseline truth ratings for each claim without any solving effects (random anagrams are solved *after* participants make a truth judgment)	(1) Providing truth ratings for all claims (2) Unscrambling all anagrams with the help of a hint, reporting aha experiences, and a 20-s delay between anagrams Aim: This condition establishes *true* baseline truth ratings, where all anagrams are solved after all truth ratings are completed

## Results

We generally used the same analytic methods as outlined in experiment 1, except that instead of using Welch’s *t*-tests to examine the between-subjects effects, we used independent samples ANOVAs with truth ratings as the DV and condition as the factor. Standardizing the difficulty and completion time resulted in similar Ns in the four conditions: *Anagram Normal* = 149, *Anagram Delay* = 157, *Anagram After Truth* = 132, and *Anagram After Everything* = 121. Based on the same preregistered exclusion criteria as Experiment 1, 329 participants were excluded for failing to solve any anagrams (*N* = 158), solving all anagrams (*N* = 23), experiencing no aha moments (*N* = 174), or experiencing aha moments on all trials (*N* = 38). As a result, 676 participants were included in analyses (see Table 1 for a breakdown of the various conditions). For hypotheses and preregistered analysis plan see https://osf.io/qmp4d, for data see https://osf.io/xug7p/.

The mean solution rate for anagrams presented alongside hints and an unfinished worldview claim was 53% (*SD* = 24%). When only the hint was provided, a similar solving rate was found, 57% (*SD* = 24%), suggesting that the hints helped to balance solving rates. We also successfully equated aha moments across the two key conditions: *Anagram Normal* condition (*M* = 44%, *SD* = 26%), *Anagram Delay* condition (*M* = 42%, *SD* = 26%). Using a multilevel regression model with aha moments as a fixed effect and participants as a random effect, we found that anagrams that elicited aha moments (in the *Anagram After Delay* and *Anagram Normal* conditions) were more likely to be correctly solved (*M* = 33%, *SD* = 24%) than those that did not (*M* = 22%, *SD* = 24%), *b* = 0.38, *p* < 0.001, *t* = 13.14, *d* = 0.65.

### Truth judgments: comparison across conditions (between)

Our key prediction was that truth judgments would be lower in the *Anagram Delay* condition than in the *Anagram Normal* condition. An independent samples ANOVA revealed a main effect of condition, *F*(3, 672) = 12.52, *p* < 0.001, *η*_*p*_^2^ = 0.053. Follow-up Tukey comparisons support our key prediction that a delay would remove the aha misattribution effect: the *Anagram Normal* condition elicited higher truth ratings (*M* = 6.94; *SD* = 2.16) than the *Anagram Delay* condition (*M* = 5.98; *SD* = 1.66), *t*(672) = 4.60, *p* < 0.001, *d* = 0.50. Moreover, the *Anagram Normal* condition elicited higher truth ratings than the *Anagram After Truth* condition (*M* = 5.97; *SD* = 1.61), *t*(672) = 5.04, *p* < 0.001, *d* = 0.51, and the *Anagram After Everything* condition (*M* = 5.90; *SD* = 1.72), *t*(672) = 5.39, *p* < 0.001, *d* = 0.53. There were no other significant effects, indicating that the order of anagram solving was redundant and that the anagram simply needed to be solved at the same moment as the worldview claim was presented.

### Truth judgments: solving and aha moments (within)

We used a multilevel regression model to test our prediction that participants in the *Anagram Normal* condition would rate claims associated with solved anagrams as more likely to be true than claims associated with unsolved anagrams, including solving as a fixed effect and participants as a random effect. We also predicted that participants in the *Anagram Normal* condition would rate claims as more likely to be true if they experienced an aha moment while solving the anagram. We further predicted that both of these effects would be absent or weaker in the *Anagram Delay* condition.

The predicted interaction between condition (Anagram Normal vs. Delay) and solved anagrams was significant, *b* = 0.42, *t* = 2.06, *p* = 0.041. Inconsistent with predictions, the higher truth ratings in the presence of solved anagrams (*M* = 7.27, *SD* = 2.56) than unsolved anagrams (*M* = 6.67, *SD* = 2.40) in the *Anagram Normal* condition failed to reach conventional levels of significance, *b* = 0.35, *t* = 1.79, *p* = 0.083, *d* = 0.09. In the *Anagram Delay* condition, no effect of solving emerged on truth ratings (*M* = 6.00, *SD* = 1.91 vs. *M* = 6.11, *SD* = 1.96), *b* = −0.18, *t* = −0.87, *p* = 0.396, *d* = −0.05.

As predicted, the interaction between condition (Anagram Normal vs. Delay) and experiencing aha moments was significant, *b* = 0.70, *t* = 3.47, *p* < 0.001. Simple effects analyses revealed that experiencing aha moments when solving anagrams resulted in higher truth ratings (*M* = 7.54, *SD* = 2.62 vs. *M* = 6.60, *SD* = 2.19) in the *Anagram Normal* condition, *b* = 0.85, *t* = 5.54, *p* < 0.001, *d* = 0.23, but not in the *Anagram Delay* condition (*M* = 6.20, *SD* = 2.36 vs. *M* = 6.02, *SD* = 1.82), *b* = 0.12, *t* = 0.76, *p* = 0.447, *d* = 0.03.

**Figure 3:**
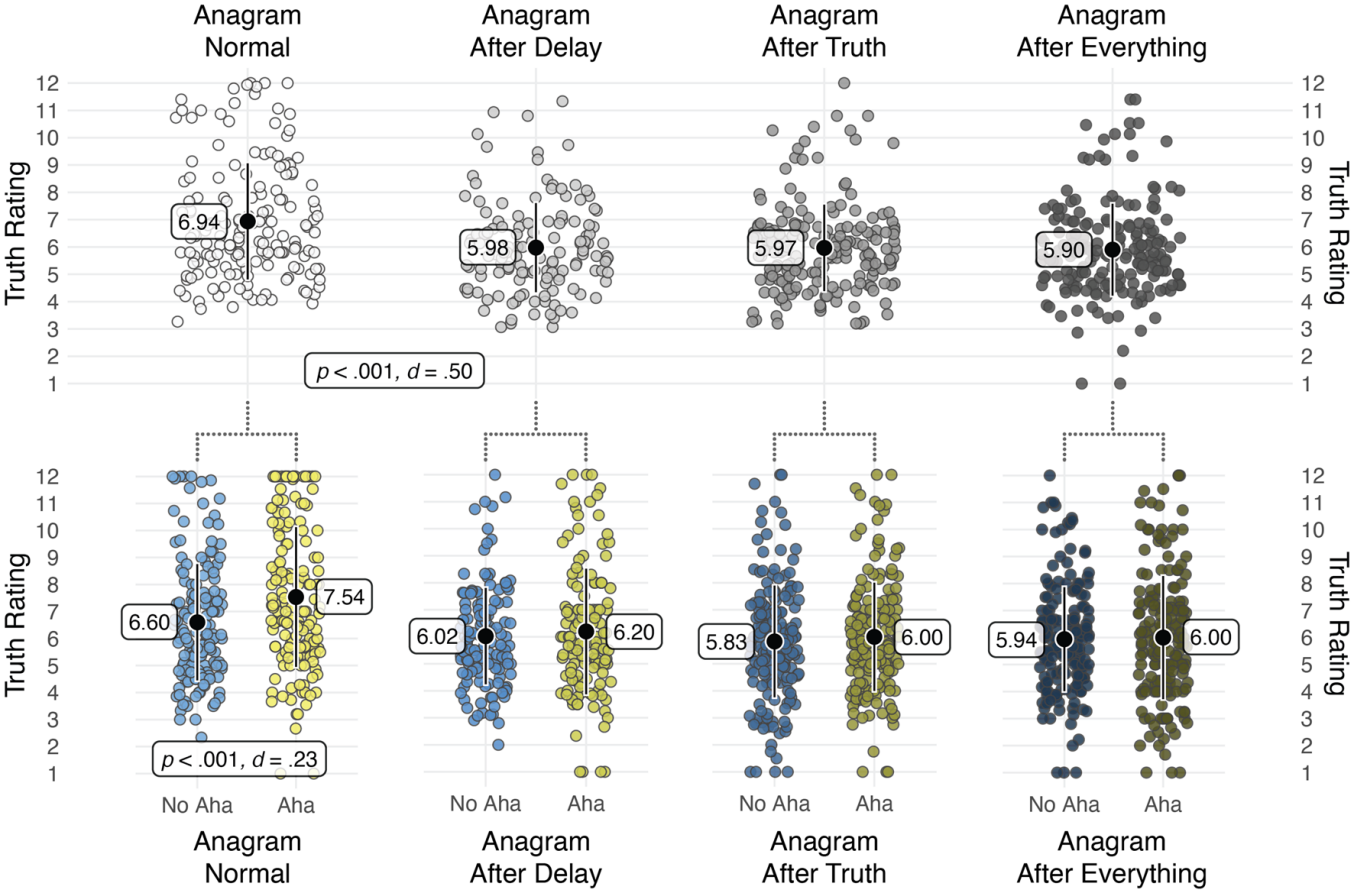
The plots in the top figure illustrate truth ratings for the four different between-subjects conditions, as well as the key analysis comparing anagrams without a delay (Anagram Normal condition) to anagrams with a delay (Anagram After Delay condition). In the bottom of the figure, the conditions are split between presence or absence of aha, illustrating an effect of aha on truth *only* when the anagram is presented at the same time as the worldview belief. Each large black dot represents the mean and black lines represent + /− 1 standard deviation. The circles represent individual participants with random horizontal jitter to aid visualization.

Finally, we conducted the aha moments analysis among the subset of correctly solved anagrams. Inconsistent with predictions, the interaction between condition and experiencing aha moments, with truth judgments as the DV, was not significant, *b* = 0.33, *t* = 1.00, *p* = 0.32. Nonetheless, simple effects analyses revealed that correctly solved anagrams accompanied by aha moments had higher truth ratings than those not accompanied by aha moments (*M* = 7.45, *SD* = 2.88 vs. *M* = 6.73, *SD* = 2.65) in the *Anagram Normal* condition, *b* = 0.58, *t* = 2.65, *p* = 0.009, *d* = 0.16 (replicating Experiment 1), but not in the *Anagram Delay* condition (*M* = 6.17, *SD* = 2.47 vs. *M* = 5.93, *SD* = 2.45), *b* = 0.23, *t* = 0.92, *p* = 0.361, *d* = 0.06. The key findings are illustrated in Fig. 3.

## Discussion

The present study tested whether incidental aha experiences could influence the perceived veracity of different worldview beliefs. In the first experiment, we found that participants rated worldview statements as truer when they had just attempted to solve anagrams corresponding to those statements. We also found that successfully solving the anagram led to higher truth ratings than failing to solve them. And finally, for the subset of correctly solved anagrams, those that elicited aha moments had the highest truth ratings of all. We then directly replicated this effect. In a second experiment, we manipulated the timing of anagram solving and therefore also aha moments. Although the interaction did not reach significance, simple effects showed that aha moments increase perceived truth only when they occur at the same time that the worldview belief is presented. In short, it seems that temporally contiguous artificially induced aha experiences can impact people’s assessments of central premises about the world, giving them a ring of truth that they would not otherwise enjoy.

The possibility that feelings of insight have an impact on one’s judgments is not itself surprising. There is a long list of domains where feelings influence decisions, including jury decision-making^35^, risk judgments^36^, truth and memory judgments^26,37,38^ and gambling and probability judgments^39^. But why would the aha experience influence truth ratings about something as seemingly unrelated and fundamental as worldview beliefs? Aha experiences are characterized by an immediate sense of confidence and pleasure in the content of an idea or solution^21,40^. This feeling of certainty is warranted—aha moments tend to correspond to accurate solutions^14,21–25^. Because aha experiences tend to be a marker of good ideas, it makes sense that humans learn to draw on this feeling as a source of information about our beliefs^9,14^. Our findings thus favor the hypothesis that aha moments are not simply epiphenomenal—like the steam whistle of an engine—but may have causal influence guiding decisions about the veracity of new ideas, like the coal that fuels the engine^9,26,29^.

It is worthwhile briefly distinguishing our aha misattribution account from fluency or ease of processing effects^37,41^. Under the fluency account, when an anagram is solved—regardless of aha moments—there ought to be an increase in fluency. However, *within* solved anagrams, the presence or absence of aha moments led to higher truth ratings, thus going above and beyond fluency alone. The fluency account would also presumably predict higher truth values when there are no anagrams to solve, as the presence of anagrams ought to lead to a more disfluent experience overall. Yet, we found the opposite in Experiment 1.

In order to align our findings within a theoretical framework, we have previously proposed that the feeling of insight may provide a meta-cognitive signal about previous learning, partially indicating the degree to which previous knowledge is consistent with the novel idea^9,14,29^. That is, the insight feels true because it is consistent with our existing models. This in turn permits the agent to meta-cognitively “trust” their feelings of insight in order to act quickly and efficiently on new ideas, like a heuristic. The logic behind our experiment was therefore that an artificially elicited feeling of insight could lead to a ‘ring of truth’ that is misattributed due to this heuristic process to the worldview presented at the same time. The heuristic framework can also be integrated within a hierarchical active inference model, where higher-levels of processing make predictions of lower-level processes^42–44^. Here, the lower-level processing occurs implicitly, and when a valuable novel idea is uncovered it is passed higher in the hierarchy leading to a sudden switch into awareness of a new idea. As the idea breaches consciousness the feeling of insight may act as a signal of expected confidence in, or “precision”^45^ of, the new idea given prior belief. Higher-order metacognitive levels may then infer given this input (new idea + insight) that a potentially good idea has been uncovered from lower-level implicit processes^46^.

The feeling of insight is hard to measure objectively. Therefore, one limitation of our study is that participants needed to report on their aha experiences after making their truth judgments, leaving room for potential confounds. Nevertheless, our supplementary analyses reveal that aha moments were present to a similar extent when the anagrams were solved separately from the truth judgments (see Appendix B), suggesting that the truth judgments were not influencing aha judgments. Worldviews with the highest average truth judgments were also associated with similar rates of aha moments as worldviews with the lowest average truth judgments, indicating that the believability of the worldview did not influence subsequent aha judgments. Finally, in Experiment 2 we included a delay condition, where participants solved anagrams then waited ten seconds before seeing the worldview and then making truth and aha judgments. Here we found no effect of aha on worldviews, consistent with the idea that the aha experience needs to happen concomitantly with the worldview and that truth statements do not affect aha moments, since this part of the trial was held constant. Another area that may demand further research is on the specific kinds of worldviews that are affected by aha experiences. We did not have a systematic criteria for selecting our worldviews except that they ought to be difficult to prove and were representative of the worldview categories outlined in previous work^30^. It remains to be seen what kinds of beliefs can be influenced by feelings of insight.

A fruitful path for future work is to investigate the possible effects of aha on decision-making both in problem-solving contexts and beyond^47,48^. Aha moments can be incorrigible^22^, difficult to forget^49^ and can promote inspiration and action^21^. It is also well known that humans often fail to introspect about the true causes of their feelings or actions^50–55^. Aha moments can mark a valuable new discovery, but if this process breaks down or is misinformed, then they may also perpetuate and entrench false beliefs. If irrelevant aha moments can influence worldview statements, how much more impactful might a relevant one be? A potentially disastrous example of this mechanism in action might be seen in the QAnon phenomenon. Here an unknown individual(s) set up vague clues for the public to identify patterns across the media, political and presidential proceedings, and other current events, with the goal of confirming a grand conspiracy in which the president of the United States was acting behind the scenes to stop a pedophilic cannibalistic cabal. The level of support for the movement is hard to measure, but appears remarkably high given the bizarre nature of the claims^56^. QAnon provides a potential real-life example of our findings in its demonstration that the way the mind constructs ‘insights’ is fallible^57^, and yet these insights can induce real and sometimes dangerous behaviour.

Moving forward, we encourage further research to establish the causal connection between aha and belief change. We also encourage a research program on the underlying mechanisms and predictors of *false* insights—circumstances and states of mind in which this usually adaptive heuristic may break down. A paradigm has recently been developed to experimentally elicit false insights^57^, which could yield valuable data for understanding the development of delusions in clinical populations. Consider, for example, the case of John Nash, the Nobel Laureate and mathematician. When he was asked why he believed he was being recruited by aliens to save the world. He said, “…the ideas I had about supernatural beings came to me the same way that my mathematical ideas did. So I took them seriously”^58^. Together with the example of Rob Sips^1^ described earlier, such anecdotes may point to the failure of an otherwise adaptive Eureka heuristic^29^. Under ordinary conditions, the feeling that accompanies a new idea reveals that inaccessible processes have yielded a valuable conclusion. This heuristic view of the aha moment also makes sense evolutionarily, as we often must decide quickly whether a new idea is good or bad; a thorough analysis is not always possible when an audience member asks a challenging question or a hungry lion is at one’s heels.

### Supplementary Information

Supplementary Information.
