# Supplementary material for: Irrelevant insights make worldviews ring true
Source: Sci Rep. 2022 Feb 8;12:2075. doi: 10.1038/s41598-022-05923-3 (PMC8826315; doi:10.1038/s41598-022-05923-3)

Appendix A

Worldview Statements and Anagrams

The table below includes the worldview statements and anagrams used in the experiments including the hints used in Experiment 2.

Table S1. List of anagrams

Statement	Anagram	Hint
Nothing that happens is truly random.	donmra	Like rolling dice.
Ants are capable of thinking.	hgniiknt	Solving problems or considering possibilities.
Free will is a powerful illusion.	oinliusl	Sleight of hand, mirage.
We gain the deepest knowledge by simply being in nature.	artenu	Forests and mountains.
It is sometimes pointless to pursue justice.	tsceuji	Right and wrong, fairness.
The key to lasting happiness is discipline.	cdeispnili	Strict dedication, control, and effort.
It is enough to simply be talented.	tenledat	Athlete, musician, artist, Kobe Bryant, innate.
Everyone has approximately the same perception of reality.	yraitle	Actual life, truth.
A society can still be considered complete without equality.	laiyeutq	Fairness, balance, Martin Luther King.
Living a good life is mostly about maximizing pleasure.	lpsereua	A positive emotion or sensation, laughter.
People's core qualities are fixed.	dxfei	The house always wins.
A person's behaviors and decisions are ultimately determined.	temrdednie	Destiny, settled before.
Human thought and behavior are not influenced by culture.	uretclu	Visiting museums.
One should always look ahead to avoid surprises.	psesusrir	Happy Birthday!, unexpected.
It is not worth it to risk the unknown.	wonunnk	Secret, darkness, never explored.

Note: In each case the anagram is a scrambled version of the last word of the statement.

Appendix B

Experiment 2: Solving and aha moments by anagram condition

The below figures illustrate solving rates (A) and aha moments (B) for the anagram normal condition and the other conditions combined. We present these frequencies to preempt a possible confound, namely that implicit belief in a particular worldview elicits more aha moments for associated anagrams (i.e., reverse causality). If reverse causality were present, then we would expect higher aha and solving rates when the worldview statement and the anagram were presented together, particularly for claims that participants tended to believe (i.e., in the anagram normal condition). However, this was not the case: The rate of aha moments in the Anagram Normal condition (M = 0.44, SD = 0.09) did not differ from that of the Anagram Delay condition (M = 0.42, SD = 0.06), $t(14)$ = 0.70, p = 0.493. Similarly, the solving rate in the Anagram Normal condition (M = 0.52, SD = 0.16) did not differ from that of the Anagram Delay condition (M = 0.57, SD = 0.15), $t(14)$ = -0.84, p = 0.409. Moreover, of the 15 anagrams, the aha rates of anagrams with the three highest truth scores (M = .41, SD = 0.09) did not differ from the aha rates of anagrams with the three lowest truth scores (M = 0.48, SD = 0.05), $t(3)$ = -1.11, p = 0.341. Similarly, the solving rates of anagrams with the three highest truth scores (M = .52, SD = 0.21) did not differ from the aha rates of anagrams with the three lowest truth scores (M = 0.66, SD = 0.13), $t(3)$= -0.97, p = 0.395.

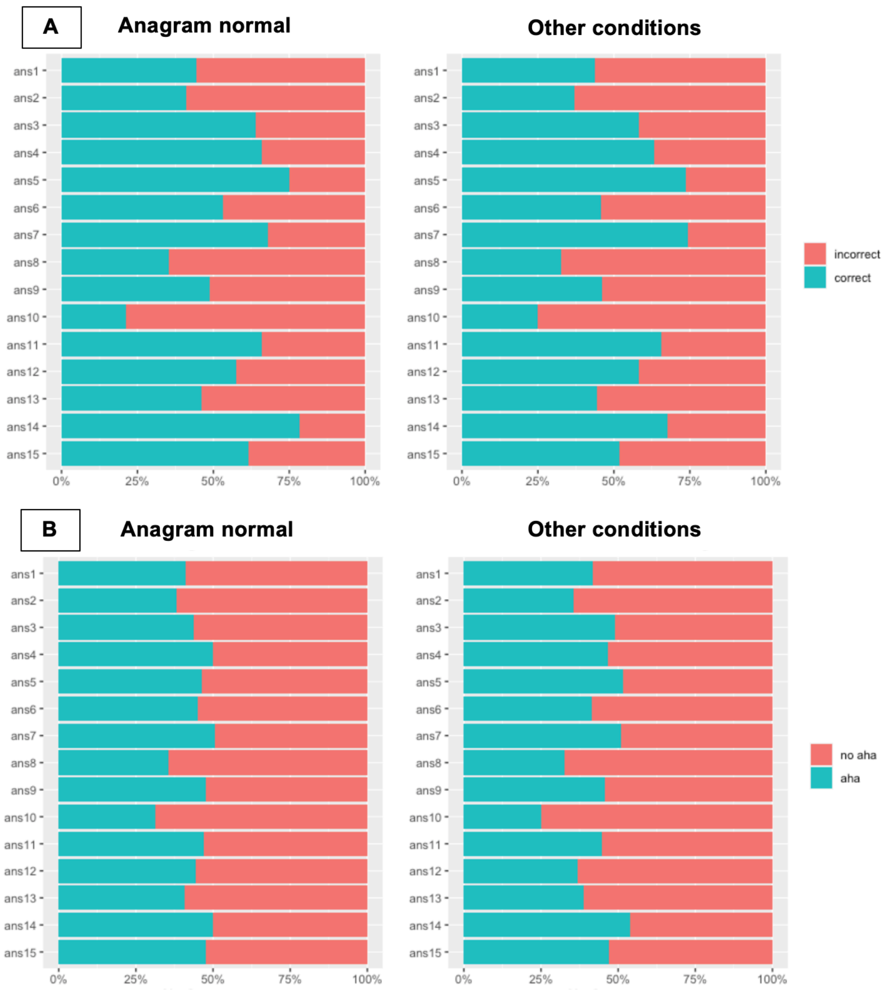

Supplement: Supplementary file 1 — Supplementary Information. [file 41598_2022_5923_MOESM1_ESM.pdf]
